# Probiotics combined with Budesonide and Ipratropium bromide for chronic obstructive pulmonary disease: A retrospective analysis

**DOI:** 10.1097/MD.0000000000037309

**Published:** 2024-03-08

**Authors:** Chen Chen, LingBo Wu, LiJun Wang, XinHeng Tang

**Affiliations:** a Department of Pulmonary and Critical Care Medicine, The Affiliated Nanhua Hospital of Hengyang Medical College, University of South China, Hengyang, China; b Department of Stomatology, The Affiliated Nanhua Hospital of Hengyang Medical College, University of South China, Hengyang, China.

**Keywords:** budesonide, chronic obstructive pulmonary disease, ipratropium bromide, probiotics

## Abstract

To explore the effect of probiotics combined with budesonide and ipratropium bromide in the treatment of chronic obstructive pulmonary disease (COPD) on lung function and gut microbiota. This was a retrospective study of prospectively collected clinical data of 118 patients with COPD admitted to our hospital between January 2020 and December 2022. According to the treatment records, 59 patients received budesonide and irpratropium bromide (control group), and 59 patients received probiotics combined with budesonide and irpratropium bromide (observation group). The lung function, inflammatory factor levels, airway remodeling, and gut microbiota before and after treatment were compared between the 2 groups. After treatment, FVC, MMEF, PEF, and FEV_1_ in the 2 groups were higher than before treatment, and the values in the observation group were higher than those in the control group (*P* < .05). After treatment, the serum levels of TNF-α, IL-6, and PCT in the 2 groups were lower than before treatment, and the levels in the observation group were lower than those in the control group (*P* < .05). After treatment, the levels of serum MMP-9, VEGF, basic fibroblast growth factor, and NGF in the 2 groups were lower than before treatment, and the levels in the observation group were lower than those in the control group (*P* < .05). After treatment, the levels of lactobacilli and bifidobacteria in the 2 groups increased compared to those before treatment, and the observation group had a higher level, while the levels of Enterobacteriaceae and Enterococcus were lower in the observation group than those before treatment (*P* < .05). Based on budesonide and irpratropium bromide, probiotic treatment of COPD is more conducive to reducing the degree of inflammatory reactions, inhibiting airway remodeling, regulating the level of gut microbiota, and promoting the recovery of lung function.

## 1. Introduction

Chronic obstructive pulmonary disease (COPD) is a common and frequent disease of the respiratory system.^[1]^ It is mainly characterized by increased airway inflammation, sustained airflow restriction, and lung tissue damage.^[1,2]^ Patients often experience varying degrees of shortness of breath, expectoration, chest tightness, cough, and difficulty breathing.^[2,3]^ If a patient does not receive timely and effective intervention, respiratory failure and/or pulmonale may occur as the disease progresses.^[1–3]^

Budesonide and irpratropium bromide are commonly used for the clinical treatment of COPD.^[4]^ Among them, budesonide is a glucocorticoid with anti-inflammatory effects, which can inhibit airway remodeling and reduce airway edema; ipratropium bromide is a new choline-soluble bronchodilator, which competitively binds M1 and M3 receptors with acetylcholine to dilate bronchi, and the anticholinergic effect lasts for a long time, which can inhibit airway remodeling and inflammatory response.^[4,5]^

With the deepening of clinical research, it has been found that gut microbiota disorders not only affect the gastrointestinal immune response, but also affect the functions of the lungs and other organs.^[6,7]^ The correlation between the gut microbiota status and respiratory diseases, such as COPD, has also been clinically confirmed.^[6–8]^ Therefore, it is believed that regulating the gut microbiota by providing probiotics is conducive to further improving the therapeutic effect of COPD.^[7,8]^ In recent 2 years, our hospital has used probiotics in combination with budesonide and irpratropium bromide to treat patients with COPD. In this study, we aimed to retrospectively analyze the prior prospectively collected data to explore the therapeutic effect of probiotics combined with budesonide and irpratropium bromide in treating patients with COPD.

## 2. Materials and methods

### 2.1. Patients

This was a retrospective study of prospectively collected clinical data of 118 patients with COPD admitted to our hospital from January 2020 to December 2022. According to the treatment records, 59 patients received budesonide and irpratropium bromide were set as the control group, and 59 patients received probiotics combined with budesonide and irpratropium bromide was assigned to the observation group. Our study was approved by the Ethics Review Board of The Affiliated Nanhua Hospital of Hengyang Medical College, University of South China (2021-KY-88).

#### 2.1.1. Inclusion criteria.

- Patients met the diagnostic criteria for COPD;^[9]^
- Patients aged between 18 and 80 years;
- Patients who received more than 14 days of treatment;
- Patients with complete clinical data.

#### 2.1.2. Exclusion criteria.

- Patients with systemic lesions such as kidney and liver;
- Patients who received glucocorticoid treatment;
- Patients with digestive system diseases;
- Patients complicated with interstitial pneumonia, pulmonary tuberculosis, or bronchiectasis;
- Patients had a history of bronchial asthma.

### 2.2. Methods

After admission, patients in both groups received routine interventions, including bronchodilation, sputum evacuation, anti-infection, and oxygen inhalation. On this basis, patients in the 2 groups were assigned different treatment plans.

Control group: Budesonide (specification:2 mL:1 mg, manufacturer: AstraZeneca Pty Ltd, H20140475) in combination with Ipratropium bromide (Specification:2.5 mL; Manufacturer: Laboratoire Union, France; Approval No.: H20150173), 2 mL Budesonide + 1 mL Ipratropium bromide + 2 mL normal saline twice a day.

Observation-group: On the basis of the control group, probiotics were administered orally with Bifidobacterium Lactobacillus triple live bacteria (Inner Mongolia Shuangqi Pharmaceutical Co., Ltd.; Approval No.: S19980004; Specification:0.5 g * 24 tablets), 3 times a day, 2 g/time.

### 2.3. Outcome measures

1. Lung function: FVC, MMEF, PEF, and FEV_1_. They were measured using the German company power cube diffusion lung function detector.
2. Inflammatory factor indicators: TNF-α, IL-6, and PCT. We extracted 5 ml of fasting venous blood, centrifuged the supernatant, and measured the levels using enzyme-linked immunosorbent assay. The reagent kit was purchased from Shanghai Enzyme-linked Biotechnology Co., Ltd.
3. Airway remodeling indicators: MMP-9, VEGF, basic fibroblast growth factor, and NGF. 5 ml of fasting venous blood was extracted, centrifuged, and measured using an enzyme-linked immunosorbent assay. The reagent kit was purchased from Shanghai Enzyme-linked Biotechnology Co., Ltd.
4. Gut microbiota indexes: Lactobacillus, Bifidobacterium, Enterobacteriaceae, and Enterococcus. 1 g of fresh feces was collected from the patient, placed in a 4°C environment for 12 hours, and then placed in a −80 °C environment. The sample was placed in a test tube (sterilized and dried), diluted 10 times with sterilized paint, and shaken for 1 minute. After 10 times gradient dilution, 0.1 ml was poured into the culture medium, and The Gut microbiota was isolated and cultured for 48 hours at room temperature. After treatment using the Gram staining method, bacterial colonies were counted on a plate.

### 2.4. Statistical analysis

Data were analyzed using the SPSS26.0 software (IBM Corp, Armonk, NY, USA). The normality of the data was evaluated using the Shapiro–Wilk test. Normally distributed data were expressed as mean ± standard deviation. The independent sample *t* test was used for inter-group comparisons, and the paired *t* test was used for intra-group comparisons. Data of non-normal distribution were expressed as median and interquartile interval. Mann–Whitney *U* test was used for inter-group comparisons, and the Wilcoxon signed-rank test was used for intra-group comparisons. Counting data were represented by the number of cases, and Chi-squared test was used for comparison between groups. Differences were considered statistically significant at *P* < .05.

## 3. Results

A total of 118 patients were enrolled in this study, including 66 males and 52 females, aged 45 to 78 years, with a median age of 64 (62, 67) years, and the course of the disease was 1 to 9 years, with a median of 5.5 (4,7) years. The clinical data of sex, age, disease course, smoking, and alcohol consumption were balanced and comparable between the 2 groups (*P* > .05) (Table 1).

**Table 1:** Comparison of 2 sets of baseline data.

Baseline data	Observation-group (n = 59)	Control-group (n = 59)	*χ^2^*/*Z*	*P* value
Gender (Male/Female)	35/24	34/25	0.706	.401
Age (yr)	65 (62, 67)	64 (62, 68)	−0.170	.865
Course of disease (yr)	6 (4, 7)	5 (4, 7)	0.428	.669
Smoking (Yes, %)	40 (67.8)	34 (57.6)	2.441	.118
Drinking (Yes, %)	33 (55.9)	31 (52.5)	0.553	.457

There was no significant difference in the lung function indicators between the 2 groups before treatment (*P* > .05). After treatment, FVC, MMEF, PEF, and FEV_1_ in the 2 groups increased compared to before treatment, and the observation group had higher values than the control group (*P* < .05) (Table 2).

**Table 2:** Comparison of lung function indicators between 2 groups.

Time	Group	FVC (L)	MMEF (L/s)	PEF (L/min)	FEV_1_ (L)
Before treatment	Observation-group (n = 59)	2.5 (2.2, 2.6)	1.2 (1.1, 1.3)	346 (336, 358)	1.6 (1.5, 1.7)
	Control-group (n = 59)	2.6 (2.3, 2.7)	1.3 (1.1, 1.3)	354 (325, 369)	1.6 (1.3, 1.7)
	Z	−1.784	−1.679	−1.070	−1.077
	*P* value	.074	.093	.285	.282
After treatment	Observation-group (n = 59)	3.20 ± 0.41*	2.8 (2.5, 3.0)*	476 (462, 488)*	2.69 ± 0.41*
	Control-group (n = 59)	2.81 ± 0.39*	2.5 (2.2, 2.6)*	440 (412, 458)*	2.30 ± 0.38*
	*t*/Z	5.294	−5.140	−4.963	−4.237
	*P* value	<.001	<.001	<.001	<.001

Compared with before treatment in this group

* *p* < 0.05.

FEV1 = forced expiratory volume in the first second, FVC = forced vital capacity, MMEF = maximum mid-expiratory flow, PEF = peak expiratory flow.

There was no significant difference in inflammatory response indicators between the 2 groups before treatment (*P* > .05). After treatment, the serum levels of TNF-α, IL-6, and PCT in the 2 groups decreased compared to before treatment, and the observation group was lower than that in the control group (*P* < .05) (Table 3).

**Table 3:** Comparison of levels of inflammatory factor indicators between 2 groups.

Time	Group	TNF-α (ng/L)	IL-6 (pg/ml)	PCT (ng/ml)
Before treatment	Observation-group (n = 59)	94 (89, 101)	25.98 ± 6.04	0.75 (0.69, 0.82)
	Control-group (n = 59)	95 (87, 101)	26.35 ± 5.66	0.81 (0.69, 0.89)
	*t*/Z	−0.413	−0.821	−1.702
	*P* value	.680	.411	.089
After treatment	Observation-group (n = 59)	56.54 ± 13.02*	13.22 ± 2.37*	0.18 ± 0.05*
	Control-group (n = 59)	69.63 ± 15.25*	16.86 ± 3.32*	0.26 ± 0.08*
	*t*	−4.689	−2.596	−5.363
	*P* value	<.001	.009	<.001

Note: Compared with before treatment in this group

* *p* < 0.05.

IL-6 = interleukin 6, PCT = procalcitonin, TNF-α = tumor necrosis factor-α.

**Figure 1.**
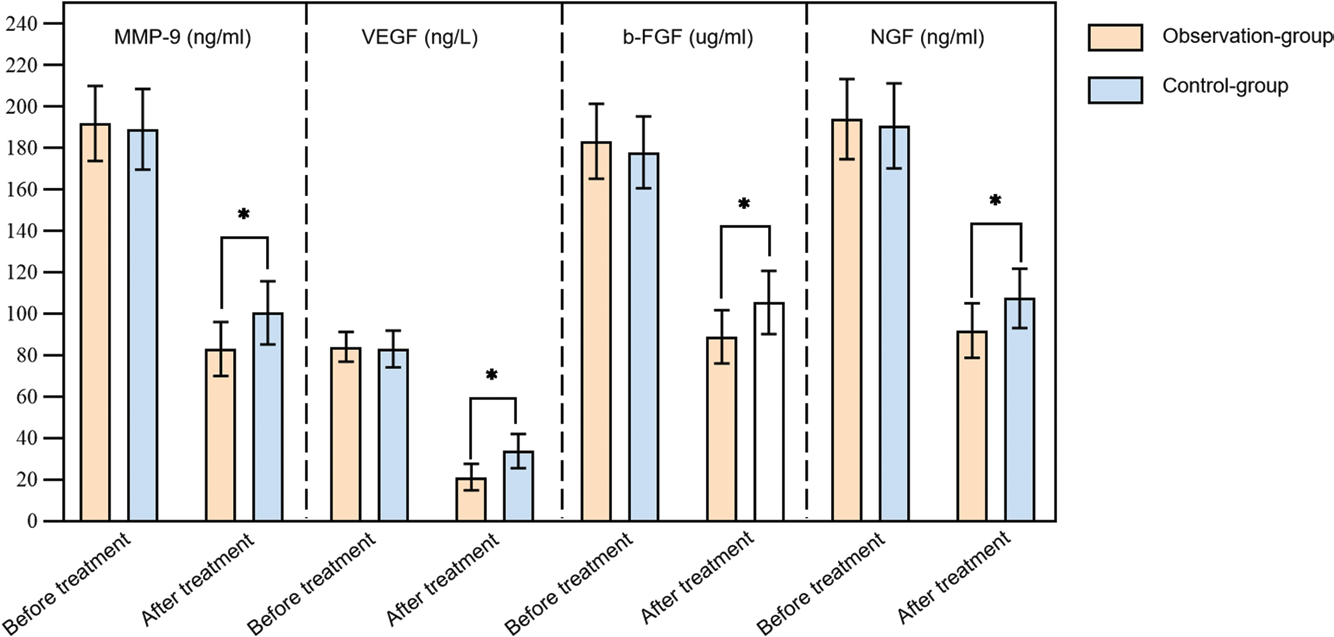
Comparison of airway remodeling indicators between 2 groups. * indicates *P* < .05. Error bars reflect standard deviation.

There was no significant difference in airway remodeling indicators between the 2 groups before treatment (*P* > .05). After treatment, the serum levels of MMP-9, VEGF, basic fibroblast growth factor, and NGF in the 2 groups decreased compared to before treatment, and the observation group was lower than that in the control group (*P* < .05) (Fig. 1).

**Figure 2.**
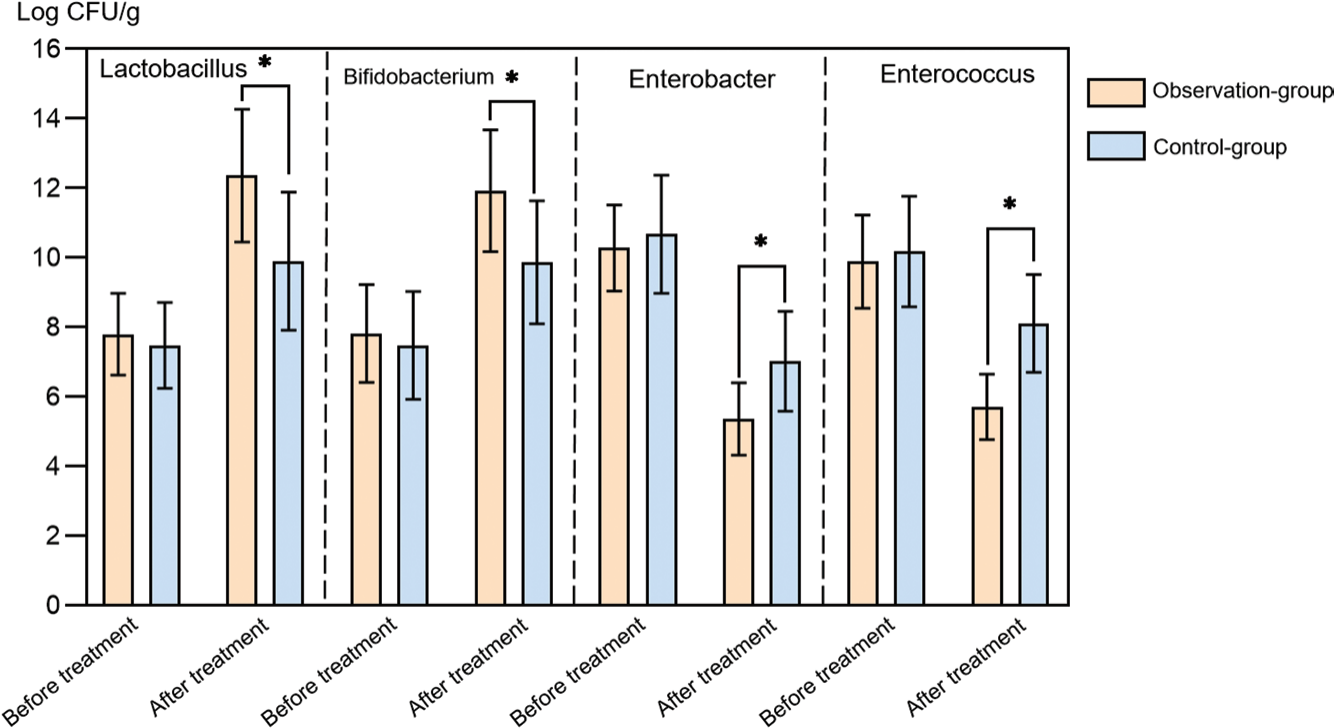
Comparison of gut microbiota indicators between the 2 groups. * indicates *P* < .05. Error bars reflect standard deviation.

There was no significant difference in the level of Gut microbiota between the 2 groups before treatment (*P* > .05). After treatment, the levels of lactobacilli and bifidobacteria in the 2 groups increased compared to before treatment, and the Observation-group had a higher level; The levels of Enterobacteriaceae and Enterococcus were lower than before treatment, and lower in the Observation-group (*P* < .05) (Fig. 2).

## 4. Discussion

Studies have pointed out that most patients with COPD have varying degrees of gut microbiota disorder, which leads to invasion of harmful bacteria and increased endotoxin production.^[10]^ Intestinal bacteria can pass through the intestinal mucosa to reach distant organs and mesenteric lymph nodes, resulting in the excessive secretion of inflammatory mediators and aggravating the disease process.^[10,11]^ Therefore, it is believed that adopting probiotics based on conventional drugs has a positive effect on the implementation of comprehensive interventions for COPD. The results of this study showed that the levels of lung function indicators in the observation group were significantly higher than those in the control group, whereas the levels of airway remodeling indicators were significantly lower than those in the control group. Probiotics have a high auxiliary application value during routine drug therapy for COPD as they can downregulate the expression level of inflammatory factors, alleviate the degree of inflammatory response, inhibit airway remodeling, and correct the disorder of gut microbiota to improve lung function.^[11,12]^ The main reason is that the combination of conventional Western medicine and probiotic treatment can correct gut microbiota disorders, inhibit inflammatory cell infiltration, alleviate pulmonary inflammation, and play an important role in reducing lung function damage and ensuring the recovery of body function.^[12,13]^ Qu L et al^[14]^ pointed out that the interaction between gut microbiota and the lungs is known as the “lung gut axis,” which can affect airway homeostasis and the immune response. The goal of preventing or treating respiratory infections can be achieved by regulating the gut microbiota. Therefore, the addition of probiotics to treat COPD has a positive significance in improving the treatment effect of the disease.^[13,14]^ It is mainly because probiotics include fecal Enterococcus, bifidobacteria, and Lactobacillus acidophilus, which can promote the colonization and large-scale reproduction of probiotics in the intestine, supplementing the normal number of bacteria in the intestine, eliminating potential pathogenic bacteria in the intestine, restoring the balance of the gastrointestinal microbiota environment, strengthening intestinal defense ability, and maintaining the integrity of the gastrointestinal structure.^[15]^ This is consistent with the results of the present study.

Inflammatory factors play important roles in the pathogenesis and progression of COPD. TNF-α, IL-6, and PCT are common clinical inflammatory factors.^[16]^ Under normal physiological conditions, their serum level is relatively low, but if inflammation or injury occurs, they will abnormally increase, so they can be used for the diagnosis and treatment evaluation of diseases.^[16,17]^ This study showed that the levels of inflammatory factors were significantly lower in the observation group than in the control group after treatment. This shows that probiotics combined with budesonide and irpratropium bromide for the treatment of COPD can help reduce the degree of inflammatory reactions in patients.^[17]^ This is mainly because probiotics can promote digestion, enhance immunity, and exert anti-inflammatory effects.^[18]^ At the same time, Enterococcus and Enterococcus are conditional pathogenic bacteria, whose excessive proliferation can cause varying degrees of damage to intestinal mucosa and trigger a series of inflammatory reactions.^[17,18]^ The supplement with exogenous probiotics can correct the disorder of the gut microbiota, restore the internal environment of the machine, and achieve anti-inflammatory effects.^[16–18]^

With the deepening of clinical research, the relationship between the gut microbiota and COPD and the value of gut microbiota-related indicators in the evaluation of disease efficacy has attracted attention. Sun et al^[19]^ confirmed that the diversity and abundance of the intestinal microbiome can affect the onset and progression of respiratory diseases, which can be used as a new treatment strategy for COPD. Wang et al^[20]^ also suggested that the disruption of the gut microbiota is closely related to the pathogenesis and progression of COPD. The clinical symptoms of patients can be relieved by conventional drug treatment; however, it is difficult to effectively regulate the status of the gut microbiota.^[19,20]^ However, supplementation with probiotics can correct the disorder of gut microbiota and increase the number of probiotics.^[20]^ This study found that the levels of gut microbiota-related indicators in the observation group were higher than those in the control group after treatment. It suggests that the addition of probiotics during the routine treatment of COPD can regulate the gut microbiota, which is conducive to ensuring a good disease prognosis.^[18,19]^ The main reason is that probiotic preparations can restore the balance of gut microbiota, inhibit the excessive aggregation of neutrophils and lymphocytes in the lungs, reduce the content of inflammatory factors, and improve lung function and respiratory status.^[18,20]^ Moreover, supplementing probiotic preparations can accelerate the proliferation of intestinal epithelial cells, downregulate intestinal mucosal permeability, inhibit the production of intestinal-derived endotoxins, promote the digestion and absorption of nutrients by individuals, and protect the intestinal mucosa, so as to achieves the therapeutic goals.^[18–20]^ A double-sided, randomized controlled study by Karim A et al^[21]^ on 104 patients with COPD from 2 tertiary hospitals receiving placebo treatment or probiotic treatment showed that the probiotic group had significantly improved exercise ability, and the levels of inflammatory factors such as C-reactive protein in the body were significantly reduced.^[21]^ It has been confirmed that the auxiliary intervention of probiotics based on symptomatic treatment of COPD can improve the muscle function, strength, and body function of COPD patients by stabilizing neuromuscular and regulating intestinal permeability.^[20,21]^ A recent meta-analysis by Pei C et al^[22]^ confirmed the important role of probiotics in alleviating clinical symptoms, improving lung function, and ensuring good disease outcomes in patients with COPD. Jamarkandi SA et al^[23]^ also confirmed that there are many risk factors that can cause gut microbiota imbalance and intestinal microecology disorder in the pathogenesis and progression of COPD, resulting in increased secretion and synthesis of endotoxins, but the above problems can be improved by the application of probiotics.

### 4.1. Limitations of the study

This is a single-center retrospective analysis with a small sample size and selection bias, which may have resulted in partial result bias. In the future, prospective studies with large sample sizes may be required to verify this conclusion.

## 5. Conclusion

Based on budesonide and ipatropium bromide, probiotic treatment of COPD can reduce the degree of inflammatory reaction, inhibit airway remodeling, regulate the level of gut microbiota, and promote the recovery of lung function. This could provide suggestions for relevant clinical treatments.

## Author contributions

**Conceptualization:** Chen Chen.

**Data curation:** LingBo Wu, Lijun Wang.

**Formal analysis:** LingBo Wu, Lijun Wang.

**Software:** LingBo Wu.

**Writing – original draft:** Chen Chen.

**Writing – review & editing:** XinHeng Tang.
